# Annotations

**Published:** 1902-11-15

**Authors:** 


### ANNOTATIONS.

### The Trail of the Skirt.

History is silent regarding Adam's views on feminine garments, but ever since the coming out of Eve's first daughters circumstantial evidence has been forthcoming to show that dress has loomed large in the thoughts and desires of the weaker sex, and has not, indeed, been without its influence in directing the evolution of mere man. And even to-day clothes, as agents for protection, adornment and topic of conversation, cannot be considered as discarded. Indeed, the subject is one which calls for intimate and expert study, and such bodies as the Rational Dress Association and the Healthy and Artistic Dress Union devote themselves to research with results which are often surprising if not actually of scientific value. The Pharos Club has recently been the scene of combat, skirts *versus* bloomers. As far as the masculine reporter can ascertain something like a compromise has been arrived at; bloomers or knickerbockers are worn, but such rationals are rendered invisible, and since what the masculine eye does not see the masculine heart does not grieve over, the skirt is allowed to cover a multitude of shortcomings. Hygienically speaking, the skirt as now prevailing stands guilty, or rather should hang condemned, of many sins. It fetters free movement, impedes progress, interferes with active exercise, produces needless fatigue, predisposes to accidents, limits the use of the hand, troubles the mind, is in constant danger of entanglements, collects dust, dirt and bacilli galore, and is oftentimes unduly expensive. Workers find it heavy, hampering, harmful and as all must admit it readily becomes filthy and unhealthy. And yet in spite of all we understand only one reformer at the recent solemn conclave appeared skirtless! We have no wish to suggest banishment for the skirt, but since the participation of women in sports and physical exercises of various kinds has clearly demonstrated the possibility of combining hygienic requirements with artistic effects, we venture to advocate a more general adoption of rational skirts. Women will gain much and man will lose nothing; the converse will also prove equally true. But on the subject of skirts woman is mistress of the situation.

### A Hint to Specialists.

The medical profession may well echo the petition of poor Burns:

"O wad some power the giftie gie us,
To see oursels as others see us."

Fortunately there is no lack of substitutes for the "power"; *Vanity Fair*, among the number, is throwing its "searchlight," and in a series of humorous articles on "Medical Men I Have Consulted" seeks to point some plain lessons in pleasant prose. In a recent number we have an interesting sketch of the heart specialist. He was "so technical in his style of conversation, and so generous in the matter of scientific terms, that I fear I was only able to afford him shadowy glimpses of the state of my inner being. Indeed, I endured so much mental strain in the endeavour to understand what he was talking about, and in politely hiding the fact that I hadn't a notion what he was alluding to. I came home quite thin and pale, and my family was really anxious about me for several days." We fear *Vanity Fair* must have sought out a very young and inexperienced "specialist"; but at all events a serious indiscretion is indicated, which some medical men are prone to be guilty of.

### The Preservation of Infants.

The progress of a nation depends in great measure on the protection of its children. The neglect of infants in this country is one of the most serious aspects of national life. Dr. T. Orme Dudfield, the Medical Officer of Health for Kensington, has recently been conducting a special investigation into this matter. He shows that the rate of infantile mortality has always been higher in Kensington than in London or England taken as a whole; in 1901 it was 161 per 1,000 and in special areas was as high as 447 per 1,000. Careful reports have been furnished by the lady inspectors. It would seem that profound ignorance prevails: various patent foods are often used for infants of two or three months, and arrowroot and cornflour are frequently administered to children under six months on the plea that the milk does not satisfy the child. Ignorance is a greater power for evil than intentional neglect or wilful unkindness. Dr. Dudfield contends that it is the duty of the State to teach the principles of health. In this matter England has need to arouse herself, or she may become a bye-word amongst civilised nations. The Medical Officer of Health for Kensington also considers that much might be accomplished by a combined effort on the part of the sanitary authority and philanthropic agencies in the establishment and maintenance of municipal crèches. Dr. Dudfield also favours the formation of a municipal milk depot, from whence infants might be provided with wholesome and suitable food.

### The Pathology of the Pawnshop.

The pawnbroker occupies a very important position in our social system. Amongst the indigent poor he may claim, like mercy, to be twice blessed, for he reaps advantage from him who gives and from those who take. In many a poverty-stricken locality the pawnshop forms the central pump in the social cardio-vascular system. It is lamentable to think that the methods and manners of not a few seem to render some such extravagant procedure an absolute necessity. But we are only concerned at the present time with the pathological aspects of the situation. There is strong evidence to show that the pawnshop readily offers a field for the transmission of morbid growths. Infectious diseases, contagious skin affections, and such communicable conditions as tuberculosis may readily be conveyed through the medium of clothing. And even when actual infectious material is not present the wearing of apparel soaked with human secretions and permeated with organic material necessarily makes for impairment of vigour and may seriously diminish the natural powers of resistance. We venture to think that medical officers of health and sanitarians generally would do well to study more carefully the flora and fauna of the pawnbroker's shop. And what we have said respecting the pawnbroker applies also in great measure to many dealers in the wonders of cast-off clothing.

